# Handgrip strength in elite youth football: potential for performance prediction and the moderating effects of age and maturation

**DOI:** 10.3389/fspor.2025.1625015

**Published:** 2025-07-08

**Authors:** Sebastian Viktor Waldemar Schulz, Lucas Wizani, Lynn Matits, Eric Schwarz, Patrick Wiedemann, Daniel Alexander Bizjak, Achim Jerg, Johannes Kirsten, Alexander-Stephan Henze

**Affiliations:** ^1^ Sports and Rehabilitation Medicine, University Hospital Ulm, Ulm, Germany; ^2^ Faculty of Sports and Health Sciences, Munich Technical University, Munich, Germany; ^3^ Clinical & Biological Psychology, Institute of Psychology and Education, Ulm University, Ulm, Germany; ^4^ Department of Sports Science, Humanities Section, Konstanz University, Konstanz, Germany

**Keywords:** biological maturation, performance testing, youth athletes, sprint speed, lower limb power, functional testing, hand grip dynamometer

## Abstract

Handgrip strength (HGS) is a simple and reliable indicator of general muscular strength, yet its relevance in elite youth football remains insufficiently understood. This study examined the utility of HGS as a practical indicator of athletic performance in this population, focusing on its associations with sport-specific motor abilities and the moderating influence of age and biological maturation. A total of 221 elite male youth football players aged 11–19 years completed a standardized performance test battery that included HGS (via dynamometer), dynamic balance (Star Excursion Balance Test), vertical jumps (Counter Movement Jump, Abalakov Jump, Heading Jump), horizontal jumps (Broad Jump, Single-Leg Hop for Distance), and sprints (10 m and 30 m). Pearson correlation coefficients were used to assess associations between HGS and motor performance outcomes, while linear regression models tested the moderating effects of age and maturity offset. HGS was strongly associated with jumping (*r* = 0.69–0.75 for vertical; *r* = 0.73–0.75 for horizontal) and sprinting performance (*r* = −0.62 to −0.73) and showed small but significant associations with dynamic balance (*r* = −0.29; all *p* < .001). Regression analyses confirmed significant main effects of HGS on jumping (*β* = 0.31–0.60) and sprinting (*β* = −0.23 to −0.33), moderated by both age and maturation status. No significant effects were observed for balance. The combination of HGS and age accounted for up to 67% of the variance in sprinting and up to 61% in jumping. These findings demonstrate that HGS is a robust and practical predictor of sprinting and jumping performance, especially when combined with age. This makes HGS a valuable, resource-efficient tool for performance diagnostics and talent development in elite and youth football, especially in settings where extensive testing is impractical.

## Introduction

Elite youth football represents a demanding and multifaceted environment in which players must meet extensive physical and physiological requirements. Match play is marked by frequent transitions between high-intensity anaerobic efforts-such as sprinting, jumping, and directional changes-and lower-intensity aerobic activities like jogging and walking (1). These demands necessitate the development of speed, strength, endurance, and agility to support repeated explosive actions and sustained performance (2). High-intensity efforts are particularly influential in goal-related situations and are often decisive for team success (3). With the ongoing evolution of the game toward greater speed and intensity, the physical performance thresholds required at elite levels have increased significantly (4).

To meet these demands, youth players in professional academy systems are immersed in structured training environments that mirror the standards of senior-level football. These systems aim to systematically enhance the physical, technical, and tactical profiles of athletes in preparation for professional competition (5). A central element in this process is the objective evaluation of individual development trajectories, considering both performance capacity and biological maturation (6, 7).

Empirical studies demonstrate that elite youth players consistently outperform their non-elite counterparts in strength, power, and speed assessments (8, 9). Widely used field-based tests include linear sprint tests (e.g., 10–30 m) to measure acceleration and top-end speed (10), as well as vertical jump assessments such as the countermovement jump and Abalakov jump to evaluate lower-limb explosive power (11). Horizontal tests like the broad jump are also commonly applied due to their relevance to sprinting and change of direction ability (12, 13).

However, the identification and progression of talent within these systems are hampered by several well-documented challenges. The relative age effect results in a selection bias favoring players born earlier in the selection year, while variations in biological maturation among age-matched athletes further complicate developmental assessments (14, 15). Early-maturing players often exhibit temporary advantages in strength, speed, and power, which may not reflect long-term potential (16). During adolescence, rapid physiological changes-particularly around peak height velocity-can lead to temporary declines in coordination and neuromuscular control, increasing susceptibility to injury (17, 18). Injury incidence rates are notably elevated in under-15 to under-17 age groups during this period of accelerated growth (19, 20). As a result, individualized monitoring and targeted injury prevention strategies have become integral components of modern academy practice (21).

Beyond performance monitoring, physical testing contributes to injury risk identification. Functional movement assessments, including evaluations of balance, flexibility, and neuromuscular control, have become central to injury prevention strategies (22, 23). The Star Excursion Balance Test is widely used to assess dynamic balance and postural stability-both of which are linked to lower-limb injury risk (24–26). Deficits identified in preseason testing can inform individualized intervention programs and reduce injury incidence during the competitive season (27, 28).

In response to these challenges, physical performance testing has emerged as a key tool in the assessment and development of youth football players. While anthropometric data and body composition measurements offer valuable structural insights, they provide limited predictive power regarding functional performance and future success (29). In contrast, sport-specific performance tests allow for the assessment of key athletic qualities and are now routinely implemented in youth academies (30).

Although comprehensive test batteries provide important data, they are often resource-intensive and time-consuming. Simpler, scalable, and accessible assessments are thus increasingly needed. One such test is hand grip strength (HGS), which offers a practical and low-cost measure of general muscular strength (22, 31). HGS testing is quick to administer, requires minimal equipment, and is suitable for a wide range of populations, including those with limited test tolerance (32).

While HGS is primarily used to assess upper-body strength, research indicates that it is also moderately associated with lower-body strength, sprinting, and jumping performance (33, 34). In athletic populations, stronger HGS values have been correlated with higher muscle mass and superior neuromuscular performance (31). In youth football, HGS has been linked to sprint speed, change of direction, and dynamic balance, especially when accounting for age and maturation status (35, 36). Furthermore, positional demands in football, such as goalkeeping and defensive duels, often require upper-body strength, reinforcing the functional relevance of HGS in this context (33).

Despite its simplicity, the role of HGS in football-specific performance diagnostics remains underexplored (22, 31). Previous studies have indicated associations between HGS and general athletic capacities, such as balance, jumping, and sprinting (33, 34, 35, 36). However, there is a lack of systematic evidence considering developmental factors, such as chronological age and biological maturation, in elite youth football. This study addresses this gap by analyzing the associations between HGS and three key sport-specific performance outcomes: dynamic balance, vertical and horizontal jumping, and sprinting. These analyses were conducted using a large, age-diverse sample of elite youth players.

Based on existing research, we hypothesize that HGS will be strongly associated with explosive motor tasks (e.g., sprinting and jumping) and that these relationships will be moderated by age and biological maturity (37–39). Furthermore, we hypothesize that HGS, particularly in combination with age, may serve as a practical predictor of performance outcomes (31, 34). Our findings aim to inform more efficient and scalable strategies for performance diagnostics and talent identification in youth football (7, 30).

## Materials and methods

### Study design

This prospective, observational, cross-sectional study was conducted in cooperation with two German elite youth football academies affiliated with professional men's teams competing in the first national and second national division. Due to the mandated gender segregation in youth football (DFB & DFL, 2022), the sample exclusively comprised male participants. Data collection took place during the annual Pre-Competition Medical Assessments (PCMAs) conducted in the pre-season period of June to September 2023. All procedures were carried out on-site at the training facilities of the respective clubs and at the Department of Sports and Rehabilitation Medicine of Ulm University Hospital. The assessments were embedded within the clubs' regular diagnostic routines and followed standardized protocols. The study was conducted in accordance with the latest version of the World Medical Association's Declaration of Helsinki—Ethical Principles for Medical Research Involving Human Subjects 2008 and approved by the ethics committee of Ulm University (No. 371/23).

### Population

The study included 221 male elite youth football players aged 11–18 years. All participants were recruited from two professional academies affiliated with first and second division clubs in the German national football league. Eligibility required enrollment in the respective academy for at least one full season, ensuring standardized exposure to elite-level training and competition. Players with chronic conditions preventing competitive football participation or whose consent was withdrawn (either by themselves or their legal guardians) were excluded. Verbal assent was obtained from all players and written informed consent from at least one parent or legal guardian was provided prior to participation.

All players competed within a structured academy framework, aligned with the national seasonal calendar (August–December and February–May). Players were assigned to age groups from Under-12 (U12) to Under-19 (U19), with training load progressively increasing with age. Training sessions followed standardized long-term development curricula implemented across both academies. U12 players trained approximately three times per week (60–75 min per session), focusing on coordination and basic technical skills. U13–U15 players trained four times per week (75–90 min), incorporating structured endurance, strength, and tactical elements. U16–U19 players trained five times per week (90–105 min), including high-intensity aerobic conditioning, strength and power sessions (twice weekly), and position-specific technical-tactical integration.

Competitive level also increased with age and development stage. Younger players (U12) typically competed in the 6th national division, U13–U15 teams in the 2nd to 3rd divisions, and U16–U19 players regularly participated in matches at the 1st or 2nd national division level. Match frequency ranged from one official league match per week (U12–U15) to one or two matches per week, including league, friendly, and international fixtures, in the older age groups. While individual training histories in total years were not assessed, the structured and age-graded progression within the academies ensured comparable developmental conditions and a consistently high level of competition across all participants.

The final sample of 221 participants had a mean age of 14.7 ± 2.3 years, a mean height of 168.7 ± 12.8 cm, a mean body mass of 57.4 ± 14.1 kg, and a mean body fat percentage of 11.1 ± 3.1%. Biological maturation was estimated using maturity offset (MO), with an average MO of 0.85 ± 2.03 years. Players reported an average training frequency of 3.6 ± 0.5 sessions per week, consistent with their respective academy schedules. Table 1 presents detailed participant characteristics and performance data.

**Table 1:** Participant characteristics and performance tests.

Test	Unit	U12	U13	U14	U15	U16	U17	U19
Age, M (SD)	[years]	11.3 (0.3)	12.5 (0.3)	13.4 (0.4)	14.6 (0.2)	15.4 (0.2)	16.4 (0.2)	17.7 (0.5)
Total players, *n*		27	29	31	31	33	32	38
Body mass, M (SD)	[kg]	38.0 (6.5)	43.0 (6.6)	49.3 (9.2)	59.5 (10.2)	64.0 (7.6)	69.2 (6.2)	71.2 (6.5)
Body height, M (SD)	[cm]	148.9 (7.3)	155.9 (7.1)	164.2 (9.2)	171.8 (7.2)	175.4 (5.9)	178.4 (5.6)	179.8 (6.7)
BMI, M (SD)	[kg/m^2^]	17.0 (1.8)	17.6 (2.1)	18.2 (2.1)	20.0 (2.3)	20.7 (1.6)	21.7 (1.5)	22.0 (1.5)
SMM/body height, M (SD)	[kg/m^2^]	7.5 (0.7)	8.2 (1.0)	8.8 (1.1)	9.2 (1.2)	9.8 (1.3)	10.2 (1.2)	10.2 (1.1)
PBF, M (SD)	[%]	11.5 (3.1)	11.2 (4.3)	11.9 (3.7)	10.5 (2.6)	10.7 (3.0)	11.3 (2.9)	10.9 (2.2)
Leg Length M (SD)	[cm]	75.0 (5.6)	80.1 (5.4)	83.7 (5.6)	88.4 (4.4)	90.0 (5.3)	91.4 (3.8)	91.4 (4.3)
Maturity Offset M (SD)	[years to pHV]	−2.3 (0.5)	−1.3 (0.5)	−0.3 (0.8)	0.9 (0.7)	1.7 (0.7)	2.6 (0.5)	3.5 (0.6)
Training frequency, *n*	[sessions per week]	3	3	3	4	4	4	4
Playing level	[national division]	4th to 5th	2nd to 3rd	2nd to 3rd	2nd to 3rd	1st to 2nd	1st to 2nd	1st to 2nd
Handgrip, M (SD)	[kg]	18.9 (4.0)	23.3 (5.2)	26.0 (6.7)	33.1 (6.1)	35.9 (5.3)	39.8 (8.4)	42.5 (7.7)
SEBT-LSI, M (SD)	[%]	105.9 (10.6)	102.3 (7.9)	101.7 (7.1)	98.6 (5.9)	97.6 (7.6)	95.9 (5.2)	97.6 (7.0)
CMJ, M (SD)	[cm]	31.8 (4.5)	32.9 (4.5)	37.1 (3.8)	40.7 (5.3)	40.5 (5.8)	42.0 (3.4)	44.7 (5.5)
AJ, M (SD)	[cm]	37.1 (4.9)	38.6 (5.7)	43.0 (4.7)	47.4 (6.1)	50.2 (6.4)	51.1 (6.6)	53.7 (5.5)
HJ, M (SD)	[cm]	38.9 (3.3)	39.8 (5.5)	46.4 (5.3)	51.0 (5.9)	50.6 (5.5)	54.0 (4.3)	56.1 (6.9)
BJ, M (SD)	[cm]	161.1 (18.0)	170.4 (16.7)	185.1 (15.7)	200.0 (17.6)	201.3 (18.0)	208.0 (16.5)	219.3 (18.3)
SLHD, M (SD)	[cm]	134.2 (14.0)	145.2 (16.9)	158.9 (12.5)	175.5 (19.4)	178.0 (15.4)	181.4 (13.0)	193.4 (14.4)
Sprint 10 m, M (SD)	[s]	2.13 (0.12)	2.02 (0.12)	1.95 (0.07)	1.86 (0.09)	1.89 (0.13)	1.79 (0.10)	1.83 (0.10)
Sprint 30 m, M (SD)	[s]	5.13 (0.28)	4.91 (0.27)	4.71 (0.21)	4.43 (0.23)	4.40 (0.25)	4.20 (0.17)	4.22 (0.12)

BMI, body mass index; SMM, skeletal muscle mass; PBF, percent body fat; PHV, peak high velocity; SEBT-LSI, star excursion balance test—limb symmetry index; CMJ, counter movement jump; AJ, Abalakov jump; HJ, heading jump; BJ, broad jump; SLHD, single-leg hop for distance. Data except for total players, trainings frequence and playing level are presented as mean (M) and standard deviation (SD); Leg length and SLHD represent the mean of left and right leg values.

### Performance test battery

Once eligibility for study was confirmed, participants underwent additional assessments of anthropometry and performance tests.

### Anthropometrics

Body mass [kg] and body fat percentage [kg/m^2^] were measured using bioelectrical impedance analysis (InBody 770, Biospace Korea, Seoul, Korea) following the standard protocol described by Kyle and colleagues (40). Standing height and sitting height were recorded using a portable stadiometer with 0.1 cm precision (Seca 213, seca GmbH & Co. KG, Hamburg, Germany). With the same precision the leg length was measured as the distance between the anterior superior iliac spine and the medial malleolus at each ankle joint (27).

### Biological maturity

To assess biological maturity, a previously validated regression equation incorporating age, body mass, leg length, standing height, and sitting height was applied. The MO was calculated as the difference between the adolescent's chronological age and their estimated age at peak height velocity (PHV), providing an indicator of biological maturity relative to peers. A positive MO value indicates that the participants have passed their peak growth phase, whereas a negative value indicated that they are pre-PHV (38).

### Handgrip strength

Handgrip strength (HGS) was measured using a hand dynamometer (SH1003, Saehan Corp., Donghae, Korea). Participants were instructed to apply maximal force to the dynamometer while maintaining a standardised posture: shoulder adducted to the body, elbow flexed at 90° (unsupported), and wrist in a neutral position (41). Participants were instructed to avoid compensatory movements that could compromise the accuracy of the test, and to ensure that the force was generated solely by the hand and forearm muscles. Each participant performed the test with both the dominant and non-dominant hand. Three measurements were recorded for each hand, and the mean value derived from the best measurements of each hand [kg] was used for analysis (42).

### Dynamic balance

To assess dynamic balance, the Star Excursion Balance Test (SEBT) was performed according to the protocols of Dominguez-Navarro and colleagues (43) and Mohammadi Nia Samakosh and colleagues (44). Participants performed the test barefoot on a marked mat (Orthelligent Screening Mat, OPED Medical Inc., Braselton, USA). They were instructed to place both hands on their hips, balance one leg, lift the other leg off the floor, extend it as far as possible to lightly touch the floor, and return to the starting position without losing balance. The SEBT was performed in three directions: anterior, posteromedial and posterolateral. Each direction was tested alternately three times per leg and the maximum reach was recorded for each trial.

To calculate a normalized composite reach score for each leg, the sum of the reach distances in the three directions (anterior, posteromedial, and posterolateral) was divided by three times the leg length and the result was multiplied by 100. This procedure was performed separately for the dominant and non-dominant leg. Finally, the Limb Symmetry Index (LSI) was used to assess the symmetry between the compound scores of the less dominant leg and the dominant leg. An LSI of 100% indicates equal performance between the legs, while values below 100% suggest a balance asymmetry (45).

### Vertical jumping tests

Vertical jump performance was assessed using three tests: the Counter Movement Jump (CMJ), the Abalakov Jump (AJ), and the football-specific Heading Jump (HJ). All tests were performed in accordance with current methodological standards for the assessment of athletic performance in adolescents (46) on a piezoelectric one-dimensional force plate (Quattro Jump, type 9290DD, Kistler, Winterthur, Switzerland), recording jump height [cm] and relative power [W/kg].

For the CMJ and AJ, participants stood upright on the platform with body weight evenly distributed between both legs. Following a verbal reference, they performed a rapid downward movement immediately followed by a maximal vertical jump. The CMJ was performed without arm movement, while the AJ included an active arm swing to enhance takeoff (47). The HJ was designed to replicate football-specific movement patterns. Players initiated the jump with a preparatory diagonal step and aimed to contact a suspended ball, simulating heading behavior under realistic game conditions. Arm movement was allowed to maintain ecological validity (48).

Each jump type was performed three times. The highest score for each trial was retained for further analysis. To minimize fatigue effects, a passive rest period of at least 2 min was observed between trials. All test procedures were performed by experienced personnel under standardized laboratory conditions to ensure high reliability and feasibility.

### Horizontal jumping tests

Horizontal jump performance was evaluated using the double-leg Broad Jump (BJ) and the Single-Leg Hop for Distance (SLHD) (49, 50).

For the BJ, participants stood with both feet parallel behind the 0.00 m mark on a floor-mounted measuring tape. For the SLHD, they positioned one foot directly behind the starting line. In both tests, participants were instructed to jump forward as far as possible. In the SLHD, they were required to land on the same leg used for take-off and maintain balance.

A trial was considered valid if the participant landed without falling, using their hands, or lifting the foot (SLHD) or feet (BJ) and maintained a stable stance for at least 3 s. The jump distance [cm] was measured from the take-off line to the nearest point of contact on landing, typically the heel. Each test was performed three times per leg (SLHD) or overall (BJ), with at least 2 min of rest between attempts. For analysis, the furthest valid distance was used for the BJ, while for the SLHD, the average of the best attempts from both legs was used.

### Sprint test

Sprint performance was assessed using a 30-m sprint test on dry artificial turf, a standard and validated method for assessing linear speed in football (3, 51). Timing was recorded using a high-precision photocell system (Witty GATE System, type WIT002, MICROGATE, Bolzano, Italy) with gates positioned at 0 m, 10 m and 30 m. The system is characterized by excellent reliability (ICC = 0.96–0.99) and accuracy <.001 s (52) (WITTY Microgate, 2024).

Participants wore football boots and initiated each sprint from a standing, staggered position. They were instructed to run with maximal effort for 30-m and completed up to three trials. To minimize fatigue, rest intervals of at least 3 min were provided between trials. The fastest sprint time across trials was retained for analysis.

### Statistics

All statistical analyses were performed using JASP (version 0.19.2; JASP Team, 2024) and R (version 4.4.1, The R Foundation of Statistical Analysis) (R Core Team, 2024). Descriptive statistics (*M*, *SD*) were calculated for all study variables. Pearson correlation coefficients were calculated to assess associations between HGS and motor performance in sport. Due to the presence of outliers in some performance measures, additional percentage bend correlations were calculated (53). As both methods gave comparable results, the results of the Pearson correlations are presented in the manuscript. The strength of the correlations was interpreted according to Cohen's guidelines (54): small (*r* = 10–0.29), medium (*r* = 0.30–0.49) and large (*r* ≥ 0.50).

To examine the potential moderating effects of age and MO on the relationship between HGS and performance scores, a series of linear regression analyses were conducted. In these models, performance scores served as the dependent variable, HGS was included as a predictor, and either age or MO was entered as a moderator (*HGS* × *Moderator*).

In addition, linear regression models were used to assess the predictive value of HGS and age on sport motor performance. Due to multicollinearity concerns (Variance Inflation Factor > 10) when both age and MO were included as predictors, age was selected as a proxy for biological development due to its greater practical relevance. Models were calculated using raw, unstandardized values to maintain applicability in practical contexts. The explanatory power of each model was assessed using the coefficient of determination (*R*^2^), with higher *R*^2^ values indicating better model fit. Statistical significance was defined as *p* ≤ .05.

## Results

Of the initial 276 participants, 55 were excluded due to missing data in anthropometric or sport-specific performance measures. Thus, the final analysis was conducted with a sample of 221 participants aged 11–18 years (Table 1).

### Correlation analyses

Table 2 displays the descriptive statistics and Pearson correlation coefficients between HGS and all performance tests. A small but statistically significant negative correlation was found between HGS and the SEBT. Significant negative correlations were also observed between HGS and sprint performance. In contrast, large positive correlations emerged between HGS and both vertical and horizontal jump performance.

**Table 2:** Descriptive statistics and correlations between HGS and performance tests.

Performance test	Unit	Mean (SD)	*r*	*p*-value
Handgrip strength	[kg]	32.1 (10.3)	—	—
Star excursion balance test—limb symmetry index	[%]	99.7 (8.0)	−0.29	<.001
Counter movement jump	[cm]	38.9 (6.5)	0.69	<.001
Abalakov jump	[cm]	46.4 (8.2)	0.75	<.001
Heading jump	[cm]	48.7 (8.2)	0.73	<.001
Broad jump	[cm]	193.9 (25.7)	0.75	<.001
Single-leg hop for distance	[cm]	168.5 (24.6)	0.73	<.001
Sprint 10 m	[s]	1.92 (0.15)	−0.62	<.001
Sprint 30 m	[s]	4.54 (0.39)	−0.73	<.001

Pearson's *r*; effect size: small [*r* = 0.10–0.29], medium [*r* = 0.30–0.49] and large [*r* ≥ 0.50], Cohen (1988) (54). The Bonferroni-corrected *p*-value based on eight tests is *p* = .00625. SD, standard deviation.

### Moderation analyses

**Figure 1:**
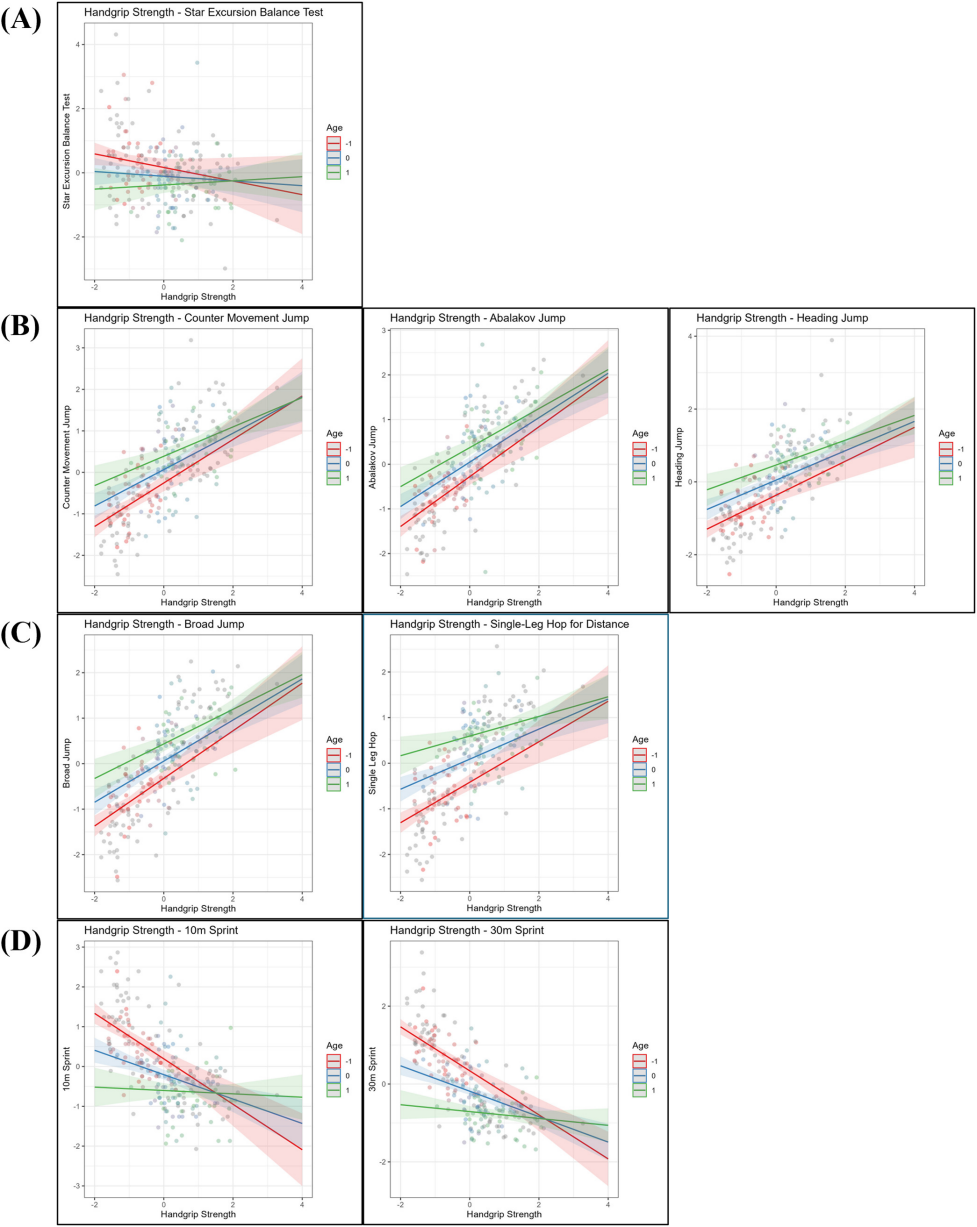
Linear regression analysis examining the relationship between handgrip strength and **(A)** dynamic balance (star excursion balance test—limb symmetry Index), **(B)** vertical jumps (countermovement jump, Abalakov jump, heading jump), **(C)** horizontal jumps (broad jump, single-Leg Hop for distance), and **(D)** sprint performance (10 m, 30 m), with age included as a moderating variable. All variables are *z*-standardized. Handgrip strength (*x*-axis) and performance outcomes (*y*-axis) are plotted at three levels of age: one standard deviation (SD) below the mean (−1 SD, red line), at the mean (blue line), and one SD above the mean (+1 SD, green line). Number of participants: *n* = 221.

**Figure 2:**
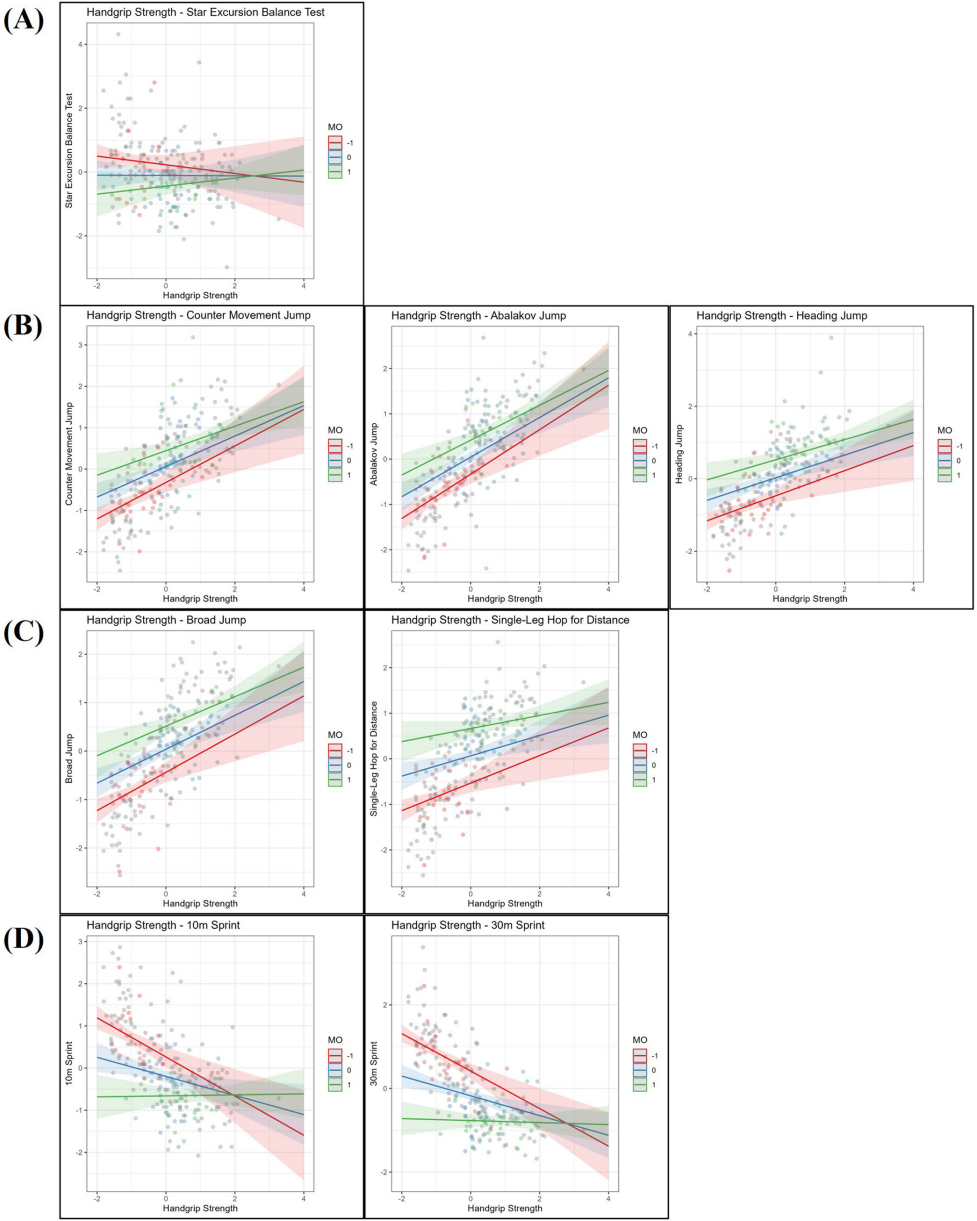
Linear regression analysis examining the relationship between handgrip strength and **(A)** dynamic balance (star excursion balance test—limb symmetry Index), **(B)** vertical jumps (countermovement jump, abalakov jump, heading jump), **(C)** horizontal jumps (broad jump, single-Leg Hop for distance), and **(D)** sprint performance (10 m, 30 m), with maturity offset (MO) included as a moderating variable. All variables are *z*-standardized. Handgrip strength (*x*-axis) and performance outcomes (*y*-axis) are plotted at three levels of MO: one standard deviation (SD) below the mean (−1 SD, red line), at the mean (blue line), and one SD above the mean (+1 SD, green line). Number of participants: *n* = 221.

This section presents the results of the moderation analyses, investigating whether age and MO independently moderate the relationship between HGS and sport-specific performance tests. For comparability, all variables were *z*-standardized. First, the moderating role of age is presented (Figures 1A–D), followed by the analyses with MO as the moderator (Figures 2A–D).

### Age

For dynamic balance performance (SEBT, Figure 1A), no significant main effect of HGS was found, but a negative main effect of age was found (*β* = −0.27, *p* = .008). Additionally, a positive interaction between HGS and age was observed (*β* = 0.14, *p* = .045), suggesting that the association between HGS and balance performance strengthened with increasing age.

Regarding vertical jump performance (Figure 1B), positive main effects of HGS were found for the countermovement jump (CMJ; *β* = 0.44, *p* < .001), the Abalakov jump (AJ; *β* = 0.50, *p* < .001), and the heading jump (HJ; *β* = 0.40, *p* < .001). Age also showed positive main effects on CMJ (*β* = 0.32, *p* < .001), AJ (*β* = 0.32, *p* < .001), and HJ performance (*β* = 0.41, *p* < .001). No significant interaction effects between HGS and age were observed for any of the vertical jumping tests.

For horizontal jump performance (Figure 1C), positive main effects of HGS were observed for the broad jump (BJ; *β* = 0.45, *p* < .001) and the single leg hop for distance (SLHD; *β* = 0.33, *p* < .001). Age also showed positive main effects on BJ (*β* = 0.38, *p* < .001) and SLHD (*β* = 0.51, *p* < .001) performance. A negative interaction between HGS and age was found for SLHD (*β* = −0.11, *p* = .01), suggesting a slight decrease in the strength of the association between HGS and SLHD with increasing age. No significant interaction effect was found for BJ.

Finally, for sprint performance (Figure 1D), negative main effects of HGS were found for both the 10 m sprint (*β* = −0.31, *p* < .001) and the 30 m sprint (*β* = −0.33, *p* < .001), indicating that higher HGS was associated with faster sprint times. Age also showed negative main effects on 10 m (*β* = −0.40, *p* < .001) and 30 m sprint performance (*β* = −0.52, *p* < .001). In addition, positive interaction effects between HGS and age were observed for both sprint distances (10m: *β* = 0.26, *p* < .001; 30 m: *β* = 0.24, *p* < .001), suggesting that the positive association between HGS and sprint performance became stronger as players aged.

### Maturity offset

For dynamic balance (SEBT, Figure 2A), no significant main effect of HGS was observed, but a negative main effect of MO was found (*β* = −0.33, *p* = .005). There was no significant interaction between HGS and MO.

For vertical jump performance (CMJ, AJ, HJ, Figure 2B), positive main effects of HGS were observed for the CMJ (*β* = 0.37, *p* < .001), AJ (*β* = 0.44, *p* < .001), and HJ (*β* = 0.31, *p* < .001). MO also showed positive main effects on CMJ (*β* = 0.38, *p* < .001), AJ (*β* = 0.37, *p* < .001), and HJ (*β* = 0.50, *p* < .001). No significant interaction effects between HGS and MO were found for any vertical jump test.

For horizontal jump performance (BJ, SLHD, Figure 2C), HGS showed positive main effects for both the BJ (*β* = 0.35, *p* < .001) and SLHD (*β* = 0.22, *p* = .003). MO also showed positive main effects for the BJ (*β* = 0.48, *p* < .001) and SLHD (*β* = 0.60, *p* < .001). No significant interaction effects between HGS and MO were found for the horizontal jumping tests.

In sprint performance (10 m and 30 m Sprint, Figure 2D), HGS showed negative main effects for the 10 m sprint (*β* = −0.23, *p* = .010) and the 30 m sprint (*β* = −0.24, *p* < .001). MO exhibited strong negative main effects for the 10 m sprint (*β* = −0.46, *p* < .001) and the 30 m Sprint (*β* = −0.59, *p* < .001). Additionally, positive interaction effects between HGS and MO were found for the 10 m sprint (*β* = 0.24, *p* < .001) and the 30 m sprint (*β* = 0.21, *p* < .001).

### Predictors of sport performance: handgrip strength and age

To evaluate the predictive value of HGS and age on sport-specific performance outcomes in elite youth football players, a series of linear regression analyses was performed. For each performance test, HGS [kg] and age [years] were included as predictors. The predictive strength of each model was assessed using the coefficient of determination (*R*^2^), representing the proportion of variance in performance explained by the two predictors.

The models showed significant predictive validity for almost all tests, except for the SEBT, where no significant prediction was observed after adjusting for age. For all other sport-specific tests—including vertical jumps, horizontal jumps, and sprint performance—HGS and age together accounted for a meaningful proportion of performance variability. Full regression coefficients, equations, and *R*^2^ values for each outcome are detailed in Table 3.

**Table 3:** Linear regression models predicting sport-specific performance based on handgrip strength and age.

Performance test	Regression equation	*R*^2^-value
Counter movement jump	15.13+0.27×HGS+1.04×Age	0.523
Abalakov jump	14.67+0.39×HGS+1.31×Age	0.610
Heading jump	14.42+0.31×HGS+1.65×Age	0.601
Broad jump	88.21+1.10×HGS+4.81×Age	0.621
Single-leg hop for distance	53.86+0.74×HGS+6.18×Age	0.636
Sprint 10 m	$2.50\text{–}3\times 10^{-3}\times \mathrm{HGS}-0.03\times \mathrm{Age}$	0.464
Sprint 30 m	6.45−0.01×HGS−0.11×Age	0.672

Regression equations and corresponding coefficients of determination (*R*^2^) for each performance test. Number of participants: *n* = 221.

## Discussion

The aim of the study was to investigate the associations between HGS and key sport-specific performance parameters—including dynamic balance, vertical and horizontal jumps, and sprinting—in a cohort of elite youth football players. This prospective, cross-sectional study included 221 male athletes aged 11–18 years from two German football academies. To our knowledge, no previous study has examined such a large sample from two elite youth football academies.

The results showed strong associations between HGS and both jumping and sprinting performance, whereas the relationship between HGS and dynamic balance was comparatively weak. In addition, the moderating effects of age and biological maturity on these associations were examined. While HGS consistently predicted better jumping and sprinting performance across age groups and maturity levels, balance performance was primarily influenced by developmental factors rather than strength. These findings underline the relevance of HGS as a general marker of neuromuscular performance in explosive tasks and highlight the need to consider age- and maturity-related influences in talent development and performance diagnostics.

The relationship between HGS and dynamic balance performance, as measured by the SEBT, was small (*r* = −0.29, *p* < .001). Most studies investigating HGS and balance have focused on older populations, with inconsistent results (55). In youth athletes, Kartal (35) reported moderate positive correlations (*r* = 0.51–0.55) between HGS and SEBT performance, whereas Muehlbauer and colleagues (56) observed only minimal associations (*r* = .01). The SEBT primarily assesses lower limb strength, proprioception and flexibility (55), which may not be adequately captured by handgrip strength alone.

In addition, anthropometric factors such as body weight may explain the small inverse relationship observed in this study. While higher body weight is often associated with greater HGS (57), it can impair dynamic balance performance (58). Despite age-related increases in muscle mass and strength during adolescence, transient declines in postural stability and neuromuscular control have been reported (59, 60), further complicating the relationship between HGS and dynamic balance.

Jumping performance was strongly associated with HGS, reinforcing the role of the HGS as an indicator of whole-body strength (31). Among the vertical jumping tasks, the AJ showed the strongest correlation (*r* = 0.75, *p* < .001), closely followed by the HJ (*r* = 0.73, *p* < .001). Both tests are likely to have larger correlations due to the involvement of arm swing and upper body dynamics, increasing the contribution of the HGS to ground reaction forces (61). In contrast, the CMJ, performed without arm swing, showed a slightly weaker correlation (*r* = 0.69, *p* < .001), highlighting the reduced role of upper body strength under these conditions.

Similar patterns have been reported in other sports populations. Hammami and colleagues (62) observed weaker correlations (*r* = 0.47–0.49) in adolescent handball players, possibly reflecting sport-specific demands that favour throwing and agility over jumping power. Debelsio and Otterson (63) reported a negative correlation (*r* = −0.41) between HGS and vertical jump performance in college football players, which reversed to a positive correlation after adjustment for BMI, highlighting the critical role of anthropometric factors in strength-performance relationships.

The horizontal jumping tasks followed a similar trend. BJ (*r* = 0.75, *p* < .001) and the SLHD (*r* = 0.73, *p* < .001) showed strong positive correlations with HGS. These results highlight the importance of HGS for forward explosive movements. Previous studies have reported different correlations depending on the athletic background: Nara and colleagues (64) found moderate correlations in male college students (*r* = 0.43), whereas Sarvaiya and Puntambekar (65) found very strong correlations in adolescent fencers (*r* = 0.84–0.86). These discrepancies suggest that sports that emphasize upper body strength may strengthen the association between HGS and horizontal jump performance.

Sprint performance over the 10 m and 30 m distances also showed large negative correlations with HGS, highlighting its role in both acceleration and speed maintenance. The 30 m sprint showed a stronger negative correlation (*r* = −0.73, *p* < .001) compared to the 10 m sprint (*r* = −0.62, *p* < .001), suggesting that HGS may contribute more to maximal sprint velocity than to initial acceleration. The initial acceleration phase is primarily dependent on lower limb explosive power and technical execution (66), whereas maintaining high velocity requires greater upper body strength for postural control and running efficiency (67). This finding is consistent with previous studies highlighting the role of whole-body strength in sprint performance in young athletes (68, 69). Although Cronin and colleagues (31) questioned the direct relevance of HGS to sprinting in field sports, several studies have documented significant negative correlations between HGS and sprint times in different populations, including male children and adolescents (37, 70–72).

### Moderating effects of age and maturity offset on the relationship between handgrip strength and performance tests

SEBT, as a parameter of dynamic balance, was significantly influenced by both age and MO, but not by HGS. Older and more biologically mature players had lower SEBT scores, likely reflecting growth-related changes in limb proportions and center of mass that temporarily impair postural stability during adolescence (60, 73). These findings are consistent with previous findings indicating superior balance control in less biologically mature athletes (74).

Interestingly, a modest positive interaction between HGS and age (*β* = 0.14, *p* = .045) suggests that greater strength may slightly attenuate the age-related decline in balance performance. As athletes progress beyond PHV, improvements in neuromuscular control may facilitate more effective integration of strength into postural stability tasks (75). In contrast, the lack of a significant interaction between HGS and MO suggests that biological maturity alone does not significantly alter this relationship. Overall, dynamic balance appears to depend more on lower limb strength, proprioceptive abilities, and flexibility than on general measures of strength such as HGS (55).

While dynamic balance performance showed only limited associations with HGS, jumping and sprinting abilities were strongly linked to strength capacities across all stages of maturation. Specifically, higher HGS was associated with better vertical (CMJ, AJ, HJ; *β* = 0.31–0.60, *p* < .001) and horizontal jump performance (BJ, SLHD; *β* = 0.33–0.60, *p* < .001), as well as faster sprint times over 10 m and 30 m (*β* = −0.23 to −0.33, *p* < .001), independent of chronological age or MO. These findings align with previous research by Pichardo and colleagues (39), who identified muscular strength as a key determinant of sprinting and jumping capacities in adolescent athletes.

Chronological age and MO independently influenced motor performance, with older and more biologically mature players performing better. This observation is consistent with previous research linking maturity-related increases in muscle mass, neuromuscular coordination, and biomechanical efficiency to improved physical performance (76, 77).

No significant interaction effects between HGS and either age or MO were observed for the vertical jumping tasks (CMJ, AJ, HJ), suggesting that the relationship between strength and jump height remains stable across different stages of maturation. Similar patterns have been reported in previous studies with children and adolescents (37).

For horizontal jumps, no interaction between HGS and age was found for the BJ. However, a small but significant negative interaction was found for the SLHD (*β* = −0.11, *p* = .01), suggesting that the contribution of HGS to unilateral jump performance may decrease slightly with increasing age. This trend likely reflects the increasing importance of technical skill and movement efficiency over pure strength (25, 26).

The strength of the association between HGS and sprint performance increased with advancing chronological age and biological maturity, as reflected by positive interaction effects for both the 10 m (*β* = 0.24, *p* < .001) and 30 m sprints (*β* = 0.21–0.26, *p* < .001). These findings suggest that HGS, as a marker of general upper-body and total-body strength, becomes increasingly important during the sprint acceleration and maximal velocity phases of adolescent maturation (67, 69, 78).

### Predicting performance tests based on handgrip strength and age

Among the various performance tests examined, HGS and age showed distinct predictive patterns. For dynamic balance performance, as assessed by the SEBT, HGS showed no significant predictive ability. This lack of association highlights the need for task-specific measures when assessing balance-related abilities, which are likely to be more dependent on flexibility, proprioception, and neuromuscular control (55). In addition, anthropometric variables such as height and body weight may have a stronger influence on SEBT performance than general strength measures (58).

In contrast, for explosive movements such as jumping and sprinting, HGS and age proved to be strong predictors. For vertical jumping, AJ (*R*^2^ = 0.610) and HJ (*R*^2^ = 0.601) had the highest predictive values, highlighting the importance of upper-body strength and coordination in jumping tasks involving arm swing dynamics. These findings are consistent with previous research suggesting that vertical jump performance is not solely dependent on lower body strength, but also on the effective interplay between upper and lower body mechanics (79). The slightly lower predictive ability observed for the CMJ (*R*^2^ = 0.523) supports this notion, as the CMJ is performed without an arm swing and relies more heavily on isolated lower-body strength and stretch-shortening cycle efficiency (80). Similar results have been reported in young basketball players, where HGS was identified as a significant predictor of vertical jump height (81).

Regression models further confirmed the predictive value of HGS and age for horizontal jump performance, with results for BJ (*R*^2^ = 0.621) and SLHD (*R*^2^ = 0.636) exceeding those observed for vertical jumps. Even higher predictive values have been reported in previous studies, such as Vaidya and Nariya (82) in college students (*R*^2^ = 0.677). Similarly, Nara et al. (64) validated the use of HGS as a predictor of horizontal jump performance in male collegiate athletes. The greater biomechanical complexity of horizontal jumps may explain their stronger association with HGS and age compared to vertical jumps. Unlike vertical jumps, which primarily involve upward forces, horizontal jumps require both vertical and forward forces, which more intensely engage the hip extensors and core stabilizers (83). Furthermore, unilateral tasks introduce additional balance and stability demands, increasing the need for neuromuscular control (25, 26). This may explain why the SLHD demonstrated slightly higher predictive power than the BJ, as greater postural stability demands place additional emphasis on strength-related skills. Additionally, the greater neuromuscular control required for single leg jumping is strongly influenced by maturational development (75), suggesting that age plays a more prominent role in predicting single leg jump performance.

Finally, HGS and age also significantly predicted sprint performance, with the 30 m sprint (*R*^2^ = 0.672) demonstrating the strongest predictive ability of all motor tests. This finding highlights the broader relevance of HGS as an indicator of whole-body strength, particularly for longer sprint distances where sustained speed and upper-body stabilization become increasingly important (69, 67). The 10 m sprint (*R*^2^ = 0.464), while still significantly predicted, had a lower predictive capacity, consistent with its greater emphasis on short-term explosiveness and technical skill rather than sustained strength over an extended sprint phase (66).

### Limitations and further research

The findings of this study were interpreted with critical consideration of the study design and current evidence, aiming to provide a differentiated understanding of the role of HGS in elite youth football.

Despite the strengths of this study, several methodological considerations must be acknowledged to appropriately interpret the findings. The cross-sectional design, with data collection at a single pre-season time point, limits the ability to capture seasonal variations in performance related to training load, fatigue, and ongoing maturation. Future research should employ longitudinal designs to track individual performance trajectories over time. Moreover, all performance tests were conducted on the same day, which may have introduced fatigue-related effects despite standardized rest periods. Although rest intervals were carefully managed, the possibility of cumulative fatigue cannot be entirely excluded. Another methodological consideration involves the use of multiple examiners due to the large sample size (*n* = 221). Although standardized protocols were followed, minor variations in instruction, encouragement, or measurement techniques may have occurred. To ensure consistency, a maximum of three different examiners were assigned per test station. Nevertheless, manual measurement of horizontal jump distances, while practical, may have introduced slight inaccuracies; future studies should consider using laser-based or force plate systems to enhance measurement precision. In addition, although the moderating effects of chronological age and MO were accounted for, HGS alone does not fully represent the complex biomechanical and neuromuscular determinants of sport motor performance. Finally, as the study sample consisted exclusively of elite male youth football players, the generalizability of the findings is limited. Future studies should examine whether the observed relationships differ by sex, competition level, age group, or sport-specific demands, and also account for potential sources of inter-individual variability in HGS values, such as hand size, hand dominance, and minor fluctuations in fatigue or motivation.

### Practical implications and recommendations for elite youth football

Based on these findings, several practical applications for elite youth football develop. The results highlight the value of HGS testing as a simple, efficient, and accessible tool for assessing strength-related abilities, particularly in settings where more comprehensive performance diagnostics may not be feasible (84). Given its associations with sprinting and lower-body, HGS offers a practical alternative for talent identification and athletic monitoring in resource-limited environments. The regression models developed in this study suggest that coaches can estimate sprint and jump performance based on HGS and chronological age. While these estimates are not intended to replace direct performance tests, they may support initial screening and serve as supplementary indicators when full testing is impractical.

From an applied perspective, HGS testing is quick (under 2 min), requires minimal equipment (e.g., a handheld dynamometer), and can be administered by a single trained coach. These characteristics make regular implementation feasible, even in environments with limited resources (84). Integrating HGS into routine assessments could support tracking neuromuscular development across different stages of maturation. As a general indicator of neuromuscular readiness, HGS reflects strength adaptations during periods of growth (85). Defining normative values according to age and maturation status would further enhance its utility by enabling coaches to compare players to developmental benchmarks and support talent identification and long-term monitoring (38).

However, it is important to note that this study did not reveal any direct implications for injury prevention based on dynamic balance testing. Given that dynamic balance performance is multifactorial and best assessed through dedicated tools such as the Y-Balance Test or dynamic postural stability measures (24, 26).

Taken together, HGS assessment has potential as a scalable and complementary tool in athlete monitoring and talent development strategies in elite youth football.

## Conclusion

This study examined the role of handgrip strength (HGS) in elite youth football. It investigated the relationship between HGS and key performance tests, the influence of biological maturation and chronological age on HGS, and HGS's predictive value for performance outcomes. HGS was strongly associated with lower-limb power, particularly sprint and jump performance, but showed only a weak relationship with dynamic balance. Regression analyses indicated that HGS, when combined with chronological age, was a strong predictor of sprinting and jumping ability. The influence of HGS on sprint performance increased with age and maturity, while its association with jump performance remained stable across maturation stages.

These findings highlight HGS as a practical, time-efficient tool for athletic profiling and talent monitoring in elite youth football, particularly where comprehensive testing is not feasible. Coaches can use HGS to track neuromuscular development and estimate performance capacities across developmental stages. To our knowledge, this is the first study to examine the predictive value and maturational interplay of HGS in relation to multiple sport-specific motor skills in elite youth football.

## Data Availability

The original contributions presented in the study are included in the article/Supplementary Material, further inquiries can be directed to the corresponding author.
